# Author Correction: Early treatment of COVID-19 with anakinra guided by soluble urokinase plasminogen receptor plasma levels: a double-blind, randomized controlled phase 3 trial

**DOI:** 10.1038/s41591-021-01569-2

**Published:** 2021-10-08

**Authors:** Evdoxia Kyriazopoulou, Garyfallia Poulakou, Haralampos Milionis, Simeon Metallidis, Georgios Adamis, Konstantinos Tsiakos, Archontoula Fragkou, Aggeliki Rapti, Christina Damoulari, Massimo Fantoni, Ioannis Kalomenidis, Georgios Chrysos, Andrea Angheben, Ilias Kainis, Zoi Alexiou, Francesco Castelli, Francesco Saverio Serino, Maria Tsilika, Petros Bakakos, Emanuele Nicastri, Vassiliki Tzavara, Evangelos Kostis, Lorenzo Dagna, Panagiotis Koufargyris, Katerina Dimakou, Spyridon Savvanis, Glykeria Tzatzagou, Maria Chini, Giulio Cavalli, Matteo Bassetti, Konstantina Katrini, Vasileios Kotsis, George Tsoukalas, Carlo Selmi, Ioannis Bliziotis, Michael Samarkos, Michael Doumas, Sofia Ktena, Aikaterini Masgala, Ilias Papanikolaou, Maria Kosmidou, Dimitra-Melia Myrodia, Aikaterini Argyraki, Chiara Simona Cardellino, Katerina Koliakou, Eleni-Ioanna Katsigianni, Vassiliki Rapti, Efthymia Giannitsioti, Antonella Cingolani, Styliani Micha, Karolina Akinosoglou, Orestis Liatsis-Douvitsas, Styliani Symbardi, Nikolaos Gatselis, Maria Mouktaroudi, Giuseppe Ippolito, Eleni Florou, Antigone Kotsaki, Mihai G. Netea, Jesper Eugen-Olsen, Miltiades Kyprianou, Periklis Panagopoulos, George N. Dalekos, Evangelos J. Giamarellos-Bourboulis

**Affiliations:** 1 4th Department of Internal Medicine, National and Kapodistrian University of Athens, Medical School, Athens, Greece; 2 3rd Department of Internal Medicine, National and Kapodistrian University of Athens, Medical School, Athens, Greece; 3 1st Department of Internal Medicine, University of Ioannina, Medical School, Ioannina, Greece; 4 1st Department of Internal Medicine, Aristotle University of Thessaloniki, Medical School, Thessaloniki, Greece; 5 1st Department of Internal Medicine, G. Gennimatas General Hospital of Athens, Athens, Greece; 6 2nd Department of Pulmonary Medicine, Sotiria General Hospital of Chest Diseases, Athens, Greece; 7 Department of Internal Medicine, Elpis General Hospital, Athens, Greece; 8 Dipartimento Scienze di Laboratorio e Infettivologiche - Fondazione Policlinico Gemelli IRCCS, Roma, Italy; 9 1st Department of Critical Care and Pulmonary Medicine, Medical School, National and Kapodistrian University of Athens, Evangelismos General Hospital, Athens, Greece; 10 2nd Department of Internal Medicine, Tzaneio General Hospital of Piraeus, Athens, Greece; 11 Department of Infectious Tropical Diseases and Microbiology, IRCSS Sacro Cuore Hospital, Negrar, Verona, Italy; 12 10th Department of Pulmonary Medicine, Sotiria General Hospital of Chest Diseases of Athens, Athens, Greece; 13 2nd Department of Internal Medicine, Thriasio General Hospital of Eleusis, Athens, Greece; 14 Spedali Civili, Brescia ASST Spedali Civili Hospital, University of Brescia, Brescia, Italy; 15 Department of Internal Medicine, Hospital of Jesolo, Jesolo, Italy; 16 1st Department of Chest Medicine, National and Kapodistrian University of Athens, Medical School, Athens, Greece; 17 Department of Internal Medicine, Spallanzani Institute of Rome, Rome, Italy; 18 1st Department of Internal Medicine, Korgialeneion-Benakeion General Hospital, Athens, Greece; 19 Department of Therapeutics, National and Kapodistrian University of Athens, Medical School, Athens, Greece; 20 Unit of Immunology, Rheumatology, Allergy and Rare Diseases (UnIRAR), IRCCS Ospedale San Raffaele & Vita-Salute San Raffaele University, Milan, Italy; 21 5th Department of Pulmonary Medicine, Sotiria General Hospital of Chest Diseases, Athens, Greece; 22 1st Department of Internal Medicine, Papageorgiou General Hospital of Thessaloniki, Thessaloniki, Greece; 23 3rd Department of Internal Medicine and Infectious Diseases Unit, Korgialeneion-Benakeion General Hospital, Athens, Greece; 24 Infectious Diseases Clinic, Ospedale Policlinico San Martino IRCCS and Department of Health Sciences, University of Genova, Genova, Italy; 25 3rd Department of Internal Medicine, Aristotle University of Thessaloniki, Medical School, Thessaloniki, Greece; 26 4th Department of Pulmonary Medicine, Sotiria General Hospital of Chest Diseases, Athens, Greece; 27 Department of Biomedical Sciences, Humanitas University, Milan, Italy & IRCCS Humanitas Research Hospital, Milan, Italy; 28 1st Department of Internal Medicine, Asklepieio General Hospital of Voula, Athens, Greece; 29 1st Department of Internal Medicine, National and Kapodistrian University of Athens, Medical School, Athens, Greece; 30 2nd Department of Propedeutic Medicine, Aristotle University of Thessaloniki, Medical School, Thessaloniki, Greece; 31 2nd Department of Internal Medicine, Konstantopouleio General Hospital, Athens, Greece; 32 Department of Pulmonary Medicine, General Hospital of Kerkyra, Corfu, Greece; 33 Department of Internal Medicine, Sotiria General Hospital of Chest Diseases, Athens, Greece; 34 Hellenic Institute for the Study of Sepsis, Athens, Greece; 35 Department of Internal Medicine, University of Patras, Rion, Greece; 36 1st Department of Internal Medicine, Thriasio General Hospital of Eleusis, Athens, Greece; 37 Department of Medicine and Research Laboratory of Internal Medicine, National Expertise Center of Greece in Autoimmune Liver Diseases, General University Hospital of Larissa, Larissa, Greece; 38 Department of Internal Medicine and Center for Infectious Diseases, Radboud University, Nijmegen, The Netherlands; 39 Department of Immunology and Metabolism, Life and Medical Sciences Institute, University of Bonn, Bonn, Germany; 40 Department of Clinical Research, Copenhagen University Hospital, Amager and Hvidovre, Denmark; 41 2nd Department of Internal Medicine, Democritus University of Thrace, Medical School, Alexandroupolis, Greece

**Keywords:** Medical research, Randomized controlled trials

Correction to: *Nature Medicine* 10.1038/s41591-021-01499-z, published online 3 September 2021.

In the version of this Article initially published, there was an error in the author affiliations. Specifically, affiliation 27, corresponding to author Carlo Selmi, has been corrected from “Humanitas Research Hospital, Milan, Italy” to read: “Department of Biomedical Sciences, Humanitas University, Milan, Italy & IRCCS Humanitas Research Hospital, Milan, Italy.”

The change has been made to the online version of the Article.

